# Evaluation of parameters in mixed male DNA profiles for the Identifiler^®^ multiplex system

**DOI:** 10.3892/ijmm.2014.1779

**Published:** 2014-05-12

**Authors:** NA HU, BIN CONG, TAO GAO, RONG HU, YI CHEN, HUI TANG, LUYAN XUE, SHUJIN LI, CHUNLING MA

**Affiliations:** 1 Hebei Key Laboratory of Forensic Medicine, Department of Forensic Medicine, Hebei Medical University, Shijiazhuang, Hebei 050017, P.R. China; 2 Institute of Statistics, Renmin University of China, Beijing 100872, P.R. China; 3 DNA Laboratory, Forensic Science Service of Beijing Public Security Bureau, Beijing 100085, P.R. China

**Keywords:** forensic genetics, short tandem repeats, male-male mixed DNA, average peak height/area of the active alleles, heterozygote balance ratio, mixture proportion, allelic drop-out

## Abstract

The analysis of complex DNA mixtures is challenging for forensic DNA testing. Accurate and sensitive methods for profiling these samples are urgently required. In this study, we developed 11 groups of mixed male DNA samples (n=297) with scientific validation of D-value [>95% of D-values ≤0.1 with average peak height (APH) of the active alleles ≤2,500 rfu]. A strong linear correlation was detected between the peak height (PH) and peak area (PA) in the curve fit using the least squares method (P<2e-16). The Kruskal-Wallis rank-sum test revealed significant differences in the heterozygote balance ratio (H_b_) at 16 short tandem repeat (STR) loci (P=0.0063) and 9 mixed gradients (P=0.02257). Locally weighted regression fitting of APH and H_b_ (inflection point at APH = 1,250 rfu) showed 92.74% of H_b_ >0.6 with the APH ≥1,250. The variation of H_b_ distribution in the different STR loci suggested the different forensic efficiencies of these loci. Allelic drop-out (ADO) correlated with the APH and mixed gradient. All ADOs had an APH of <1,000 rfu, and the number of ADO increased when the APH of mixed DNA profiles gradually decreased. These results strongly suggest that calibration parameters should be introduced to correct the deviation in the APH at each STR locus during the analysis of mixed DNA samples.

## Introduction

In forensic analysis, mixed DNA samples are composed of genetic material from more than one donor, and complex DNA mixtures involve 3 or more individuals (1,2). During criminal investigations, specimens of blood, semen, secreted fluids, excretions and epithelial cell samples are often mixed, particularly in cases of vaginal rape, anal rape and oral sodomy. Complex mixtures of DNA, such as mixed semen collected in cases of gang rape or mixed blood samples in homicide cases, are the most challenging to analyze (3–7). The contemporary analysis of mixed DNA samples often yields low detection rates that are not useful in criminal investigations. Some results collected using these methods do not meet the legal standards of relevant court systems (2). Therefore, accurate and sensitive methods for profiling mixed DNA samples are urgently required in forensic DNA testing.

Mixed DNA evidence from semen and vaginal secretions are commonly submitted for laboratory analysis in sexual assault cases. The DNA Commission of the International Society of Forensic Genetics (ISFG) published standardized unrestricted and restricted combinatorial methods for interpreting mixed male-female DNA profiles in 2006 (2). In general, unrestricted combinatorial approaches involve probability analyses of allelic bands on a short tandem repeat (STR) locus based on the separation of allele peaks by size, discounting stochastic effects that lead to the substantial imbalance of two alleles at a given heterozygous locus (2). As a result, quantitative information, such as peak height (PH) and peak area (PA) are not considered in the calculation of likelihood ratios (LRs), which limits the accuracy of these methods.

In order to achieve improved accuracy, a number of studies have reported the use of restricted combinatorial methods that require quantitative parameters (1,2,8–16), such as the heterozygote balance ratio (H_b_) and mixture proportion (M_x_) (2) in the comparison of victim DNA profiles with filtered genotype combinations. In practice, however, the restricted combinatorial methods are complicated by the presence of stutter peaks (8,11,17), drop-outs and drop-ins (2,18–25), and low-template DNA (LT-DNA) (8,11,14,24–35). Preferential amplification may occur in low-molecular-weight (LMW) alleles during PCR when H_b_ >1 (2), resulting in lower accuracy. The mixed DNA profiles are further complicated by the effects of stutter-affected heterozygotes (SAH) and allele masking, which leads to considerably more complex H_b_ regularity. Mixed DNA samples involving very small or degenerated samples are particularly prone to distortion by random effects associated with the mutual inhibition of PCR invariant DNA components, distortion of the peak balance in the PCR system, operational issues and equipment errors (10,36,37). These factors significantly increase the difficulty of scientific analysis and the interpretation of accurate results obtained from mixed DNA profiles.

For mixed male-female DNA, forensic sex-typing is generally conducted with commercial STR kits that apply the primers suggested in the study by Sullivan *et al* and the sex-typing marker, amelogenin (AMEL−) (38). Results produce characteristic male X and Y chromosome peaks that are easily distinguished from the single female X chromosome peak, although anomalous results have been reported due to abnormalities, such as primer binding-site mutations and chromosomal deletions (39). In this DNA profile, the intensity of the X peak is much higher than that of the Y peak, indicating heterozygote imbalance (i.e., H_b_ <0.6) (2). A number of studies have applied this methodology to identify the M_x_ of male-female individuals (8–11,14,37). Notably, complex mixtures involving mixed male DNA, such as mixed semen, all present 2 X/Y peaks, causing heterozygote balance (H_b_>0.6) that prohibits the estimation of M_x_ between DNA components using the AMEL locus. Therefore, the establishment of a more reliable and accurate method for the interpretation of mixed male DNA requires careful parameter selection and evaluation.

In the current study, the data distribution and statistical analysis of each parameter were carried out specifically for mixed male DNA. Using this experimental model, a relative fluorescence intensity (unit: rfu) range that provides the optimal distribution of D-value, H_b_ and allelic drop-out (ADO) according to average PHs (APHs) was determined. Mixed male DNA profiles should first meet these ‘rfu’ levels to ensure the accuracy and reliability interpretation. It should then be determined whether calibration parameters are required to correct the APH deviation during mixed DNA analysis.

## Materials and methods

### DNA sample collection

Forty anti-coagulated blood samples collected from unrelated healthy males (5 ml each) were supplied by the Blood Center of Hebei Province, Shijiazhuang, China.

### Experimental design

For our study purposes, DNA was extracted from the 40 whole blood samples, and quantified by the Applied Biosystems (Foster City, CA, USA) 7500 real-time PCR system (Life Technologies Inc., Carlsbad, CA, USA). Single DNA samples with concentration differences of <0.5 ng/μl were paired to construct a simulated mixed male DNA experimental model of 2 individuals. Gradient ratios between 2 individual DNA samples were achieved by adjusting the dilution and volume of each sample that was added to the model. In addition, the concentration of the simulated mixed DNA stock solutions were all adjusted to the desired levels within the working solution concentration range of 0.5–1.25 ng/μl, so as to meet the requirements for the DNA template of the testing kit.

### Establishment of mixed male DNA model

#### DNA extraction

Genomic DNA was extracted from the 40 male whole blood samples using the Invitrogen^®^ PureLink™ Genomic DNA Mini kit (Life Technologies Inc.). A total of 20 μl of each DNA stock solution (numbered 1–40) was diluted with nuclease-free water (Ambion, Carlsbad, CA, USA) to a final volume of 200 μl. The stock solutions of Promega^®^ 9948 Male DNA and 2800M Control DNA standards (Promega Corp., Madison, WI, USA; concentration of 10 ng/μl; volume, 25 μl) were diluted with Ambion^®^ nuclease-free water, to a final volume of 250 μl.

#### DNA quantification

DNA quantification was performed using the Quantifiler^®^ Human DNA Quantification kit (Life Technologies Inc.) containing DNA standard solution (200 ng/μl), Quantifiler Human Primer mix, and Quantifiler PCR Reaction Mix. Human Primer Mix (10.5 μl/sample) and PCR Reaction Mix (12.5 μl/sample) were mixed and then dispensed into reaction wells (23 μl each) followed by the addition of 2 μl of sample or standard to each well, to obtain a 25-μl PCR reaction system. DNA quantification was repeated 3 times for each sample, and the mean result served as the final DNA concentration.

#### Identifiler^®^ PCR and electrophoresis

With 25 μl PCR system (containing 10.5 μl of PCR reaction mix, 5.5 μl of Identifiler^®^ Primer Set, 0.5 μl of Gold^®^ DNA polymerase, 9.0 μl of nuclease-free water and 1 μl of DNA template), Identifiler^®^ PCR amplification was performed as follows: pre-denaturation at 95°C for 11 min, followed by 28 cycles of denaturation at 94°C for 1 min, annealing at 59°C for 1 min and extension at 72°C for 1 min, and a final extension step at 60°C for 60 min. The PCR products were then examined using a 10-μl electrophoresis system containing 0.25 μl of GeneScan™ 500 LIZ^®^ Size Standard, 9.25 μl of Hi-DiTM formamide and 0.5 μl of PCR product orthe AmpFlSTR^®^ Identifiler^®^ allelic ladder. Capillary electrophoresis was performed on an ABI3130xl Genetic Analyzer (Applied Biosystems).

### Parameters of mixed DNA profiles

#### APH/average PA (APA) of the active alleles

In a mixed DNA profile obtained by STR analysis, the height of the y-axis corresponding to the band of an allele is termed as the PH. The area surrounded by the x-axis and the peak outline is termed as the PA. Both allele PH and PA are expressed as relative fluorescence intensity (unit: rfu). APH or average PA (APA) is defined as the mean of PHs or PAs from all loci (excluding drop-out) in a DNA profile. The parameters, such as M_x_, H_b_ and stutter ratio fluctuate with alterations in APH or APA. APH or APA often serves as a quantitative parameter to evaluate the distribution regularity in mixed DNA analysis.

#### Analysis of M_x_

M_x_ is the proportion of a single DNA component in mixed DNA. The original proportion (theoretical M_x_) is usually not unknown in an undefined sample. Instead, PHs or PAs of the allelic bands measured in a STR locus are used for the calculation (*M_x_^l^*). The mean M_x_ is calculated as the average of the *M_x_^l^* from all loci.

#### Analysis of D-value

The D-value indicates the error between the M_x_ values calculated using the PH or PA of a locus (M_x_^l^) in a mixed-DNA profile and the mean M_x_ value of all loci in the mixed DNA profile, which is calculated using the following formula: D = |*M_x_^l^* - *M̄_x_*|

For the defined mixed DNA experimental model, the mean M_x_ can be replaced by the theoretical M_x_ (hybrid gradient designed in the model), and the following formula is used to estimate the D-value: D = |*M_x_^l^* - *M_x_*|

The D-value reflects the difference between the actual and theoretical M_x_ value of a locus in a mixed DNA profile. A low D-value indicates an accurate quantitative result of a mixed DNA experimental model.

#### Analysis of H_b_

The H_b_ is calculated as the ratio between PHs (measured as fluorescent intensities ϕ, units: rfu) of the lower peak and higher peak in the same locus, H_b_ = ϕ_a_/ϕ_b_. In the case that all DNA template amounts are >500 pg and not degraded, H_b_ >0.6, defined as heterozygote balance, usually indicates that both alleles originate from one the heterozygote of one individual. H_b_ <0.6 is defined as heterozygote imbalance, indicating that the alleles are from different individuals.

Given that the H_b_ cannot be estimated in the locus with ADO, the H_b_ is only estimated in loci without drop-out and allele sharing. The H_b_ of the mixed DNA profile is only calculated with genotypes of AB:CD or AB:CC without allele sharing. Two H_b_ values (H_b_ of AB and CD) for type AB:CD, and one H_b_ value (H_b_ of AB) for type AB:CC, as well as the corresponding numbers of APH values can be calculated.

#### Analysis of ADO

The low level of a specific DNA content may cause relative fluorescence intensity which is too low, and which cannota be separated from the background, and therefore results in the loss of an allelic peak, presenting a false homozygote. ADO can be of a single allele, two alleles, and low-copy-number mixed DNA allele.

ADO is primarily caused by a very low PH value. The total number of drop-out alleles in a DNA profile correlates with the APH of a DNA sample. The APH is defined as the mean APH of loci without drop-out in a DNA profile.

#### Statistical analysis

Locally weighted polynomial regression is a non-parametric regression method. It does not require the hypothesis of data distribution. Instead it describes the relationship of variables according to the morphology of the data. This method is more robust than the conventional least squares regression model.

Kernel density estimation is a non-parametric method to estimate a density curve driven by data distribution. The Kruskal-Wallis rank sum test is also a non-parametric test that does not depend on normal distribution of data. These provide more reliable results when analyzing non-normal distributed data compared to variance analysis.

All graphics were made using the R (version 3.0.1) software package ggplot2 (version 0.9.3).

## Results

### Establishment of simulated mixed male DNA experimental model

Based on the difference in DNA concentration (≤0.5 ng/μl), 40 male DNA samples (No. 1-40) and the Promega male DNA standard were paired. A total of 22 single eligible DNA were prepared into 11 groups of 2 mixed male DNA samples. The Promega 2800M male DNA standard working sample was further diluted to a final concentration of 0.243 ng/μl for pairing. The DNA concentrations of the 11 groups are presented in Table I. A total of 9 hybrid gradients were designed in each group of mixed male DNA samples, in triplicate for each gradient, resulting in a total of 297 samples (Table II).

### DNA quantity of mixed male DNA

The DNA quantity of the 297 simulated mixed male DNA samples was examined by assessing the selected samples using the ABI 7500 real-time PCR system. The quantification of each sample was repeated 3 times, and the mean values were taken as the DNA concentration (Table III).

Given that the concentration of the DNA template recommended by the Identifiler kit used in this study was 0.5–1.25 ng/μl, 99 mixed male DNA working solutions (11 groups of mixed DNA at 9 hybrid gradients) were diluted accordingly. Each 2-μl aliquot was diluted by 10- or 15-fold with nuclease-free water (Ambion). The 9948 and 2800M DNA standards with a concentration of <0.5 ng/μl were left undiluted. Additionally, the volume of DNA template for the NAN11 mixed DNA samples (n=27) corresponding to the male-male DNA standards was 2 and 1 μl for the other groups, including single DNA samples used for mixed male DNA PCR system (Table IV).

### D-value analysis of the experimental model

**Figure 1:**
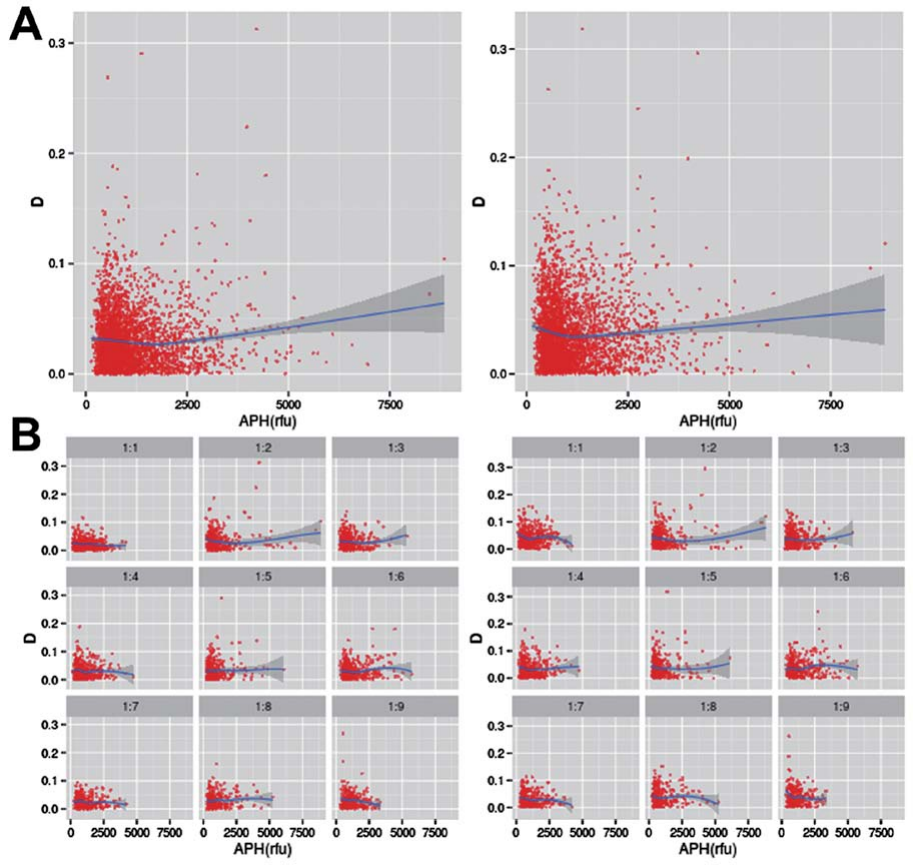
(A) Correlation between the average peak height (APH) of the active alleles and D-value; and and (B) separated analysis for the 9 mixed gradients. Left panel, D = |*M_x_^l^* - *M̄_x_*|; right panel, D = |*M_x_^l^* - *M_x_*|.

Fig. 1A illustrates the correlation between the APH and D-value of each locus in the mixed DNA profiles estimated using the 2 formulas: D = |*M_x_^l^* - *M̄_x_*| and D = |*M_x_^l^* - *M_x_*||. Of the total D-values estimated, 99.88% of the D-values estimated using the first formula were ≤0.2, and 98.25% of them were ≤0.1. While 99.88% of the D-values estimated using the 2nd formula were ≤0.2, 95.7% of them were ≤0.1. Both methods achieved 95% D ≤0.1, indicating that the error between the measured and theoretical M_x_ value of each locus in the mixed DNA profile was ≤10%. PCR amplification did not result in great alterations in the M_x_ value, indicating the reliability of PCR-based mixed DNA analysis.

The correlation between the APH and D-value of the mixed DNA samples is presented in Fig. 1B. A similar tendency in D-value distribution was observed in the 9 mixed gradients. A D-value >0.2 was found only in the gradients of 1:2, 1:5, 1:6 and 1:9, while most D-values were ≤0.1 with an APH ≤2,500 rfu. These results demonstrate a minor error between the measured and theoretical M_x_ value of each locus, suggesting that the mixed male DNA experimental models meet the requirements of scientific and rational mixed DNA analysis.

### Correlation between PH and PA

**Figure 2:**
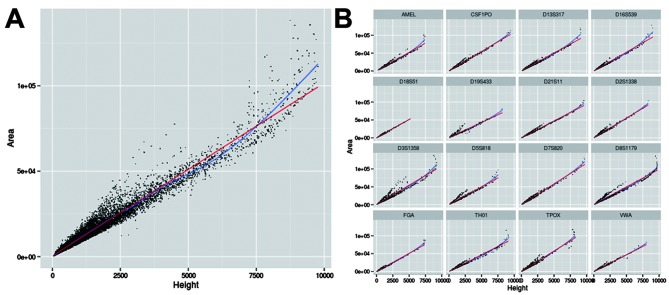
(A) Linear correlation between peak height and peak area and (B) separate analysis for the 16 short tandem repeat (STR) loci. The blue line indicates the curve fitted with the generalized additive model, and the red line indicates the curve fitted with the least squares method.

The correlation between PH and PA in the mixed DNA profile is shown in Fig. 2A. The point of inflection in the curve (blue line) fitted with the generalized additive model was present at PH of 6,250 rfu, PH increase accelerated at ≥ 6,250 rfu. The following regression equation was obtained from the curve (red line) fitted with the least squares method:

$$\text{Area}=10.12\;\text{height }(0.01825)\;(<2\text{e}-16^{***})+439.48\;(39,04147)\;(<2\text{e}-16^{***})$$

For which R^2^ = 0.9588 and the P-value was <2e-16, indicating a strong linear correlation between the PH and PA.

For the 16 STR loci analyzed (Fig. 2B), loci D19S433, D3S1358, D58S18 and D8S1179 presented a relatively weaker linear correlation between PH and PA. The other 12 STR loci showed strong linear correlation between PH and PA. These results are basically consistent with the conclusion drawn in the study by Tvedebrink *et al* (40) that there is a strong linear correlation between PH and PA. Generally, for all 16 STR loci, linear correlations between PH and PA were detected. Therefore both PH and PA may be used for the quantitative analysis of mixed DNA samples, without apparent differences in efficacy.

### Correlation between APH and H_b_

**Figure 3:**
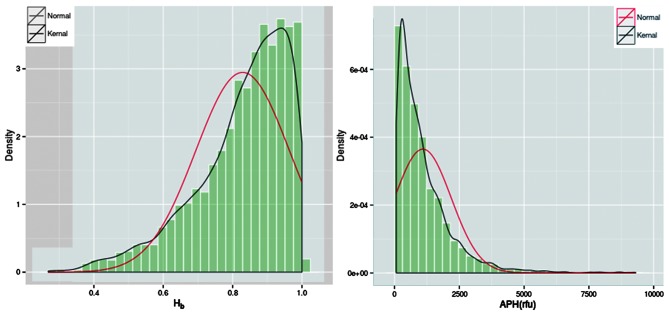
Histogram of the heterozygote balance ratio (H_b_) and corresponding average peak height (APH) at 16 short tandem repeat (STR) loci. The black lines indicate the curves fitted using the kernel density estimation, and the red lines indicate the curves fitted with normal distribution.

After the locus with drop-out was excluded, 2,535 H_b_ values and the corresponding APH values were calculated in the 297 mixed male DNA profiles. Both the H_b_ and APH showed skewed distribution (Fig. 3).

**Figure 4:**
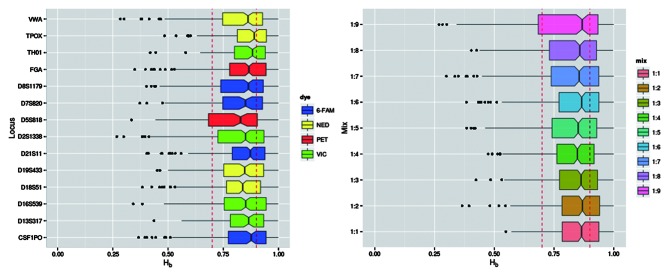
Distribution of the histogram of the heterozygote balance ratio (H_b_) at 16 short tandem repeat (STR) loci (left panel) and at 9 mixed gradients (right panel). The 4 fluorescence-labeled loci are differentiated by using 4 different colors; the red dotted lines indicate H_b_ = 0.7 and 0.9; the groove interval in the right box-whisker plot indicates the 95% confidence interval of the median.

Tables V and VI show the percentages of H_b_ >0.6 and >0.7 at 16 STR loci in the 9 mixed gradients. Fig. 4 illustrates the data distribution of H_b_ at each locus and mixed gradient, in which the red dotted lines indicate H_b_ = 0.7 and =0.9. Allele sharing at loci D3S1358 and AMEL resulted in the lack of H_b_ estimation; therefore, the statistical analysis of the H_b_ values was only carried out for 14 STR loci. It was found that the median H_b_ was higher at loci TPOX, TH01 and D21S11, with most H_b_ ≥ 0.9. The median H_b_ was lowest at locus D5S818. H_b_ distribution fluctuated greatly at loci D5S818 and D2S1338. There was little difference in the median H_b_ distribution for various mixed gradients (apart from gradient 1:8 and 1:9). The Kruskal-Wallis rank sum test revealed significant differences in the distribution of H_b_ at 16 STR loci (P=0.0063) and at 9 mixed gradients (P=0.02257).

**Figure 5:**
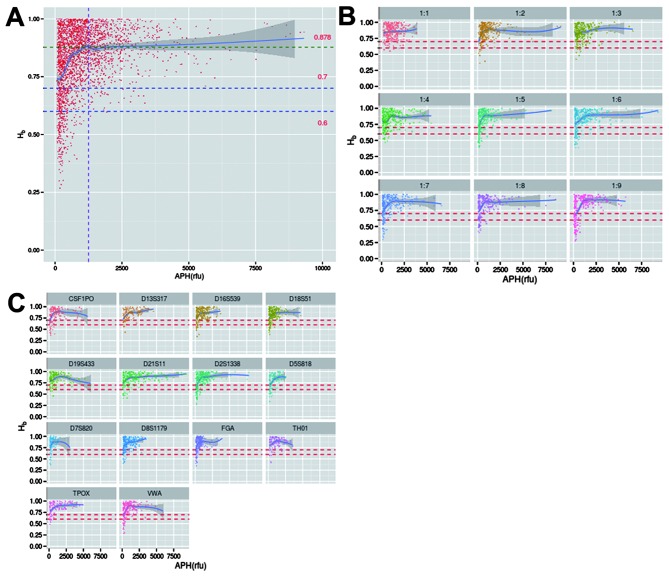
(A) Correlation between average peak height (APH) of the active alleles and 2,535 histogram of heterozygote balance ratio (H_b_) values in male-male mixed DNA profiles and (B) separated analysis of the 9 mixed gradients or (C) for the 16 short tandem repeat (STR) loci. (A) The horizontal blue dotted lines indicate H_b_ = 0.6 and 0.7; the vertical blue dotted line indicates APH = 1,250 rfu; the horizontal green dotted line indicates the mean H_b_ of 0.878 corresponding to APH >1,250 rfu; (B and C) The red dotted lines indicate H_b_ = 0.6 and 0.7.

Fig. 5A presents the correlation between the 2,535 H_b_ values and the corresponding APH in the mixed male DNA profiles. The blue solid line was plotted by using the locally weighted regression, while the grey region indicates the corresponding confidential interval. An inflection point was presented at APH = 1,250 rfu in the curve fitted by non-parametric regression. When the APH was <1,250 rfu, the H_b_ value varied between 0.75 and 0.87. In cases when the APH was ≥1,250 rfu, the H_b_ value was almost stable. The green dotted line indicates the mean H_b_ value of 0.878 corresponding to APH >1,250 rfu. It was found that 92.66% of the H_b_ values were >0.6 and 83.04% of the H_b_ values were >0.7. When the APH was ≥ 1,250 rfu, 92.74% of the H_b_ values were >0.6.

Fig. 5B and C show the changing tendency of the APH and H_b_ at the 9 mixed gradients and 16 loci fitted by the locally weighted regression, in which the 2 red dotted lines indicate H_b_ = 0.6 and = 0.7. Fig. 5B illustrates that >90% of the H_b_ values were >0.6 at gradients from 1:1 to 1:8, while 82% of the H_b_ values were >0.6 at gradient 1:9. The percentage of high H_b_ value and high APH was greater at mixed gradients of 1:2, 1:6, 1:7 and 1:8 than the other gradients. Fig. 5C illustrates that >90% of the H_b_ values were >0.6 at loci apart from D2S1338 and D5S818; moreover, the percentage of high H_b_ value and high APH was greater at loci CSF1PO, D19S433, D21S11, D2S1338 and vWA than the other loci, while APH was almost concentrated at <2,500 rfu at other loci.

### Correlation between APH and ADO

**Figure 6:**
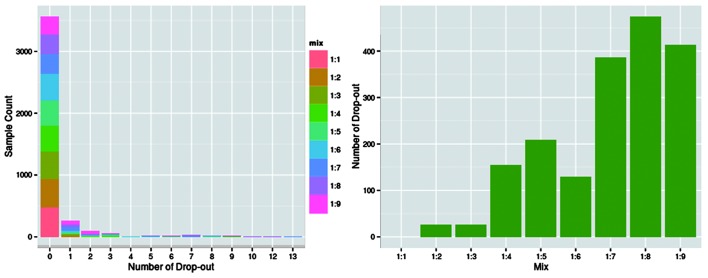
Data distribution and number of drop-out alleles at the 9 mixed gradients. The 9 gradients are marked with different colors.

**Figure 7:**
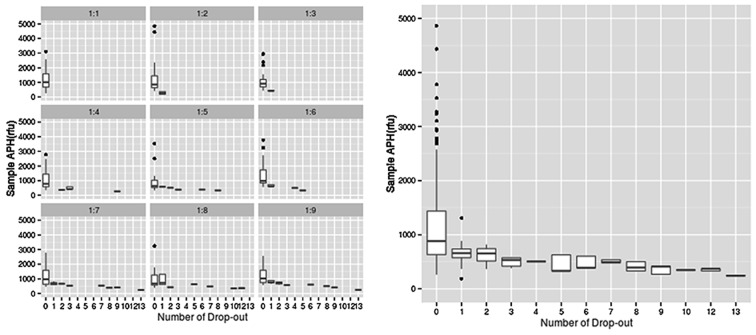
Number of drop-out alleles and distribution of average peak height (APH) in the 9 mixed gradients (left panel) and in all the mixed DNA samples (right panel).

Table VII presents 52 drop-out DNA samples in the 297 mixed male DNA profiles, with corresponding 218 ADOs. The statistical analysis of the drop-out samples distribution at the 9 mixed gradients showed that few ADOs were observed at gradients from 1:1 to 1:3, while the number of ADOs increased sharply at gradients from 1:7 to 1:9 (Fig. 6). These results demonstrated that the number of ADO correlated with the M_x_ value in the mixed DNA profiles. The incidence of drop-out would be greatly increased in an extremely imbalanced gradient (e.g., 1:7–1:9). Fig. 7 shows that many ADOs were present at gradients from 1:5 to 1:9, with a corresponding APH of <1,000 rfu (Fig. 7, left panel), while Fig. 7 (right panel) shows a wide coverage of the APH without drop-out, with the highest median detected. The gross tendency appeared to be a gradual drop in APH is accompanied by an increase in the number of ADOs.

## Discussion

The parameters analysis of this experimental model revealed a close linear correlation between PH and PA, 2 quantitative parameters of mixed DNA analysis. These results are in agreement with the conclusion drawn in the study by Tvedebrink *et al* (40). The Kruskal-Wallis rank sum test revealed differences in the H_b_ distribution at 16 STR loci and the 9 mixed gradients. The changing tendency in APH and H_b_ fitted by locally weighted regression showed a difference in the H_b_ distribution at various STR loci, suggesting different efficiencies of these loci in the mixed DNA analysis. ADO correlated with both APH and mixed gradient, and all APH drop-out values were <1,000 rfu. All results indicated that APH affects H_b_ and drop-out distribution, and H_b_ correlates with the STR locus and mixed gradients. Further studies are required to investigate the causes responsible for the variation in the forensic efficiency of Identifiler^®^ STR loci, including the different fluorescence sensitivity of genetic analyzer, which may cause APH distortion; and parameter analysis of the inter-locus balance (C_i_) on various STR loci, to reduce the bias in mixed DNA analysis.

Although stringent criteria are not generally necessary for single-DNA testing, our findings suggest that mixed STR profiles analyses should meet certain rfu levels in order to ensure the accuracy and reliability of the interpretation. The results from our study suggest that the forensic efficiency of the STR multiplex we used should be firstly evaluated in mixed DNA analysis, and calibration parameters should be introduced to correct the APH deviation of the STR loci during mixed DNA analysis.

## Figures and Tables

**Table I:** DNA concentration of the 11 groups of male-male mixed samples.

Sample no.	No. 1	Concentration (ng/μl)	No. 2	Concentration (ng/μl)	Difference (ng/μl)
NAN1	1	5.80	3	5.75	0.05
NAN2	11	7.20	40	7.21	0.01
NAN3	11	7.20	14	7.22	0.02
NAN4	20	9.24	26	9.35	0.11
NAN5	12	6.58	19	6.68	0.10
NAN6	24	5.46	27	5.48	0.02
NAN7	4	6.92	15	6.98	0.06
AN8	7	6.13	29	6.22	0.09
NAN9	11	7.20	38	7.14	0.06
NAN10	37	6.40	39	6.39	0.01
AN11	9948	0.223	2800M	0.243	0.02

**Table II:** Mixed gradient of simulated male-male mixed DNA.

Two-male mixed DNA		Mixed gradient								

	Person	1:1	1:2	1:3	1:4	1:5	1:6	1:7	1:8	1:9
Volume (μl)	No. 1	5	4	3	2	2	2	2	2	2
	No. 2	5	8	9	8	10	12	14	16	18

**Table III:** DNA quantity of the male-male mixed samples at gradient 1:9.

Sample no.	Mixed DNA quantity (ng/μl)	No. 1	Concentration (ng/μl)	No. 2	Concentration (ng/μl)	Difference (ng/μl)
NAN1	8.32	1	5.80	3	5.75	2.57
NAN2	10.94	11	7.20	40	7.21	3.74
NAN3	10.51	11	7.20	14	7.22	3.31
NAN4	13.38	20	9.24	26	9.35	4.14
NAN5	9.60	12	6.58	19	6.68	3.02
NAN6	7.01	24	5.46	27	5.48	1.55
NAN7	9.62	4	6.92	15	6.98	2.70
NAN8	8.68	7	6.13	29	6.22	2.55
NAN9	10.38	11	7.20	38	7.14	3.24
NAN10	9.31	37	6.40	39	6.39	2.92
NAN11	0.337	9948	0.223	2800M	0.243	0.114

**Table IV:** Dilution of male-male mixed DNA working solutions.^a^

Sample no.	DNA quantity (ng/μl)	Dilution factor	PCR template (μl)
NAN1	8.32	10-fold	1
NAN2	10.94	10-fold	1
NAN3	10.51	10-fold	1
NAN4	13.38	15-fold	1
NAN5	9.60	10-fold	1
NAN6	7.01	10-fold	1
NAN7	9.62	10-fold	1
NAN8	8.68	10-fold	1
NAN9	10.38	10-fold	1
NAN10	9.31	10-fold	1
NAN11	0.337	1-fold	2

a Desired concentration of DNA template, 0.5–1.25 ng/μl.

**Table V:** Percentages of H_b_ >0.6 and >0.7 at 14 STR loci.

Locus	CSF1PO	D13S317	D16S539	D18S51	D19S433	D21S11	D2S1338
H_b_ >0.6	0.91	0.97	0.94	0.92	0.96	0.95	0.88
H_b_ >0.7	0.82	0.87	0.86	0.84	0.83	0.86	0.77

Locus	D5S818	D7S820	D8S1179	FGA	TH01	TPOX	vWA

H_b_ >0.6	0.88	0.91	0.95	0.93	0.96	0.94	0.90
H_b_ >0.7	0.73	0.80	0.82	0.85	0.89	0.89	0.83

H_b_, heterozygote balance ratio; STR, short tandem repeat.

**Table VI:** Percentages of H_b_ >0.6 and >0.7 at the 9 mixed gradients.

	Mixed gradient								
	
	1:1	1:2	1:3	1:4	1:5	1:6	1:7	1:8	1:9
H_b_ >0.6	0.98	0.97	0.97	0.96	0.92	0.91	0.90	0.89	0.82
H_b_ >0.7	0.93	0.89	0.87	0.84	0.80	0.82	0.80	0.77	0.73

**Table VII:** Number of drop-out alleles in the 297 male-male mixed DNA profiles.

No. of drop-out alleles	0	1	2	3	4	5	6	7	8	9	10	12	13
No. of samples	245	19	8	5	1	2	2	3	3	3	2	2	2
